# Magnetic detection of albuminuria using hematite nanorods synthesized via chemical hydrothermal method

**DOI:** 10.22038/ijbms.2021.53918.12120

**Published:** 2021-07

**Authors:** Ali Ramzannezhad, Amir Hayati, Ali Bahari, Hamed Najafi-Ashtiani

**Affiliations:** 1 Department of Science, Faculty of Imam Mohammad Bagher, Mazandaran Branch, Technical and Vocational University, Sari, Iran; 2 Department of Physics, Faculty of Basic Sciences, University of Mazandaran,Sari, Iran; 3 Department of Physics, Faculty of Science, Velayat University, Iranshahr, Iran

**Keywords:** Albumin, Hematite nanorods, Hydrothermal method, Kidney disease, Magnetic detection

## Abstract

**Objective(s):**

Albuminuria is a biomarker in the diagnosis of kidney disease which is due to the presence of high albumin in the urine and is one of the complications of diabetes. In recent years, the methods used to identify albuminuria have been expensive and time-consuming. Furthermore, another problem is the lack of accurate measurement of albuminuria. This problem leads to kidney isolation as well as a decrease in erythropoietin levels. Therefore, the main aim of our work is to design a magnetic nanobiosensor with better sensitivity to detect minimal levels of albuminuria.

**Materials and Methods:**

In the present work, we synthesized Hematite Nano Rods (HNRs) using FeCl_3_, NaOH and Cetyltrimethylammonium bromide (CTAB) precursors via the hydrothermal method. Then, HNRs were characterized using UV-vis spectroscopy, Fourier Transform Infrared Spectroscopy (FTIR), Transmission Electron Microscopy (TEM) and Vibrating Sample Magnetometer (VSM) techniques.

**Results:**

The obtained results from clinical performance of the HNR nanobiosensor show that the magnetization changes of HNR in interaction with the albumin biomarker can determine the presence or absence of protein in biological samples. The accuracy and repeatability of the HNR nanobiosensor from the value of the R2 coefficient in the standard equation is 0.9743.

**Conclusion:**

We obtained the standard curve through interaction of the HNRs with albumin protein. The standard equation is obtained by plotting the magnetization curve of a non-interacting sample to interacting samples in terms of protein concentration. The Bland-Altman statistical graph prove that the HNR nanobiosensor is as reliable as experimental methods.

## Introduction

Conventional methods for the diagnosis of diseases (1-3) have displayed disadvantages. That is mainly because they can be quite unhelpful in the first stage of the disease, expensive, and not easily accessible to many people (4-10). A biomarker is generally referred to as a measurable indicator of some biological condition (11-20) or substance whose presence indicates the existence of a living organism (21-26). Nevertheless, a biomarker is an indicator of a biological state of protein and DNA/RNA or organic chemicals caused by abnormal cells. Nanotechnology can create unique abilities for sensing biomarkers, not possible in the past (27-29). This diagnostic method can be based on optical (30), mechanical (31), electrical (32), and magnetic (33) nanobiosensors. Many people are suffering from chronic kidney disorder but are unaware of their status. Since early symptoms of the problem seem to be minor, it is possible that people live with these symptoms for several years, but the symptoms can, unfortunately, lead to kidney failure. In the present work, we succeeded in designing a magnetic nanobiosensor by hematite nanorod for the first time with higher sensitivity than laboratory methods for measuring albumin in urine to detect kidney diseases. Hematite Nano Rods (HNRs) were synthesized by the hydrothermal method. As nanorods can interact with proteins in biological samples due to CTABs acting as a surfactant agent on the surface of nanorods, it was possible to find the amount of protein in samples by reducing the magnetization of nanorods. Magnetic nanobiosensors would be more sensitive if the minimum amount of albumin in the urine was detected. The results showed that the designed magnetic nanobiosensor with fast, accurate, and more efficient diagnosis would prevent much healthcare cost. This cost-effective and accurate diagnostic method with high sensitivity could also detect the first signals of the disease. 

## Materials and Methods

***Chemical***

The precursors used in the present work are: FeCl_3_ powder, NaOH solution, Cetyl trimethylammonium bromide (CTAB) white powder, Phosphate-buffered saline (PBS), and bovine serum albumin (BSA) powder, all bought from Sigma-Aldrich. 

***Experimental***

To synthesize hematite nanorods, we mixed 8.5 ml of sodium hydroxide (2 mol l ^-1^) with Iron (ш) chloride (0.25 mol l ^-1^). Then, 8 ml of distilled water was added to the solution. Cetyl trimethyl ammonium bromide (CTAB) was prepared at the desired concentrations. We combined the two containers together and transferred them to an autoclave (150 °C for 6 hr). Hematite nanorods were obtained by washing with distilled water. In the final step, we made interaction samples by dissolving the PBS in distilled water. Our desired concentrations were prepared in the standard range from 0.4 to 6.4 mg/ml (34, 35). All experiments were done in accordance with the Institutional Guide for the Care and Use of Laboratory samples (Ethical code, IR.UMZ.REC.1400.017). 

***Material characterization***

*UV-visible spectroscopy*

Optical properties of HNR were evaluated using the UV–Vis spectroscopy (GBC Cintra 101) technique. Distilled water as a dispersing medium and dilution process of samples was 50%. We adjusted the wavelength range from 200 to 800 nm through the scanning process.

*Transmission electron microscopy (TEM)*

We examined the morphology of the synthesized solution samples through the Transmission Electron Microscopy (TEM, Philips CM30, 150 kV) technique. 

*Vibrating sample magnetometer (VSM)*

Synthesized samples for the Vibrating Sample Magnetometer (VSM, MDKFT) technique were prepared in powder form by the freeze dryer method.

## Results

*Comparison of statistical results of the in vitro method and magnetic nanobiosensor for albumin detection*

We compared the amount of albuminuria with an *in vivo* method and magnetic nanobiosensor by collecting the urine of 6 patients. Urine samples were collected at Tooba Clinic Center, Sari, Iran. We used a statistical and comparative graph to evaluate and calculate the results of the amount of albumin in human serum, as previously described by Boland Alteman (36).

*Uv-vis spectroscopy of HNR*

**Figure 1:**
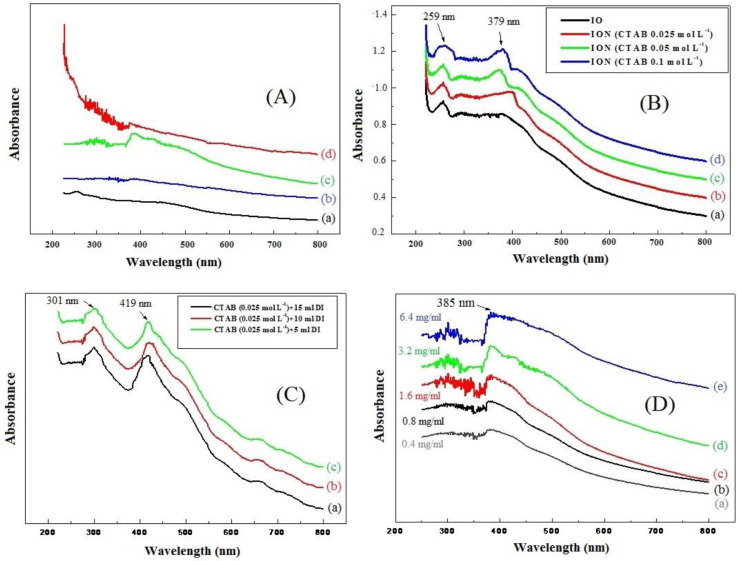
(A): UV–vis spectra of (a) Albumin, (b) Albumin-CTAB-ION, (c) Albumin-ION, and (d) Albumin-CTAB

Uv-vis spectroscopy of HNR in the presence of surfactant agent and albumin was studied within the wavelength range of 215–800 nm (Figure 1A). Absorption of hematite nanorods has two major peaks that were mostly located at 280–300 nm and 360–380 nm. There were two kinds of field transitions associated with the Fe^3+^ ligand. The first and second peaks relate to 4P and 4D ligand transition, respectively. Figure 1B shows UV-Vis spectroscopy of HNR fabricated at different molarities of CTAB at RT. CTAB as a surfactant agent could affect the shape of hematite nanostructures, causing the products to reach nanorod morphology. The as-prepared sample (without CTAB) showed the formation of the nanoparticle structure (Figure 1B (a)) due to one peak being located at 250 nm and 200–800 nm wavelength. However, for other molarities of CTAB (0.025, 0.05, and 0.1, mol l ^-1^), HNR has an absorption peak in the Uv-vis spectroscopy that was mainly observed between 210–250 nm and 350–380 nm wavelengths (Figure 1B, b-d). Therefore, it was concluded that CTAB could influence the formation of HNR. On the other hand, constant molarity of surfactant agent and distilled water resulted in nanorods with more regular shapes (Figure 1C). As can be seen, there are two sharp peaks around 250 and 420 nm in the visible region indicating that the product was able to exhibit the rod shape of hematite nanostructures. However, the peak located around 300 nm disappeared once HNRs interacted with albumin in the range of identification, and the peak at 400 nm showed a redshift after adding albumin at 0.4, 0.8, 1.6, 3.2, and 6.4 mg per ml concentration (Figure 1D, b-f). Thus, the preliminary results of UV-Vis spectroscopy showed that the interaction could change the structure of HNR due to the presence of a peak around 400 nm and hematite nanostructures having a spherical shape. Simultaneously, the TEM technique was used to confirm the above results.

*TEM of HNR*

**Figure 2:**
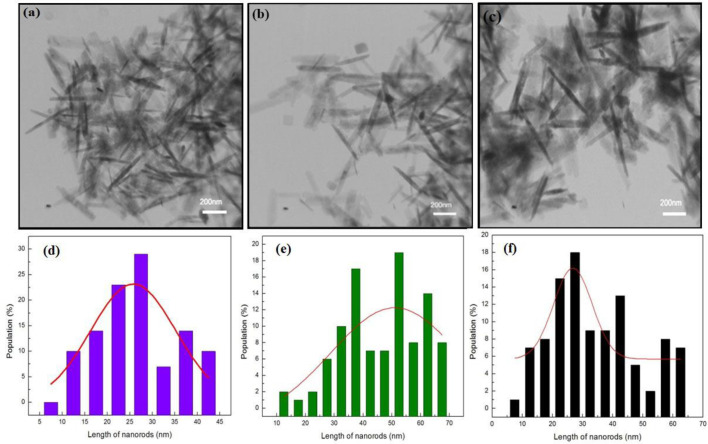
TEM images of HNRs prepared with (a) 0.025 mol L^-1^, (b) 0.05 mol L^-1^, and (c) 0.1 mol L^-1^ of CTAB on a scale of 200, 100, and 60 nm. The size distribution of HNRs prepared with (d) 0.025 mol L^-1^, (e) 0.05 mol L^-1^, and (f) 0.1 mol L^-1^ of CTAB

TEM: Transmission electron microscopy; HNRs: Hematite nano rods; CTAB: Cetyl trimethylammonium bromide

We studied the morphology of HNRs before and after modes of interaction with albumin. Figure 2 (a, b, c) shows TEM images of HNRs prepared in different molarities of CTAB. The results showed the formation of nanorod structures. The average length of HNRs in TEM images was calculated using Digimizer software. The distribution graph of nanorods is presented in Figure 2 (d, e, f). The average lengths of HNRs were about 25–30, 50–55, and 25–30 nm in different molarities of CTAB, respectively. The hematite nanorods were surrounded by CTABs that have a positive charge and albumin in the mean state including negative charge. Therefore, the only interaction between nanorods and proteins was electrostatic forces. Since the nanorods were made with CTAB as surfactant, when the concentration of protein was greater than CTAB, self-assemblies and micelles were formed. In this accumulation, the hydrophobic was in the center of the micelle and polar groups interact with the surface of the water. The size and shape of the micelle depend on the surface activation molecule structure and their spatial arrangement which are scattered in spherical form throughout the solvent. Electrostatic interactions between albumin and CTAB were tolerable to nanorods to a certain extent. In other words, HNRs would lose their rod-shaped morphology when protein concentrations were more than CTAB. Consequently, it was expected that HNRs be spherical after interaction with albumin. The method generally determined the protein’s carrying capacity with HNRs.

**Figure 3:**
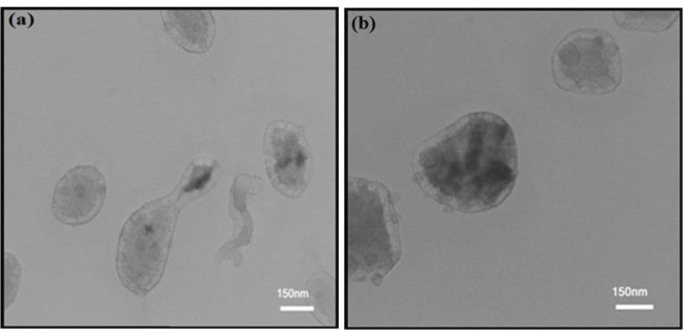
TEM images from spherical shape of HNRs prepared with (a) 3.2 mg/ml and (b) 6.4 mg/ml of albumin on scales of 150, 100, and 60 nm

**Figure 4:**
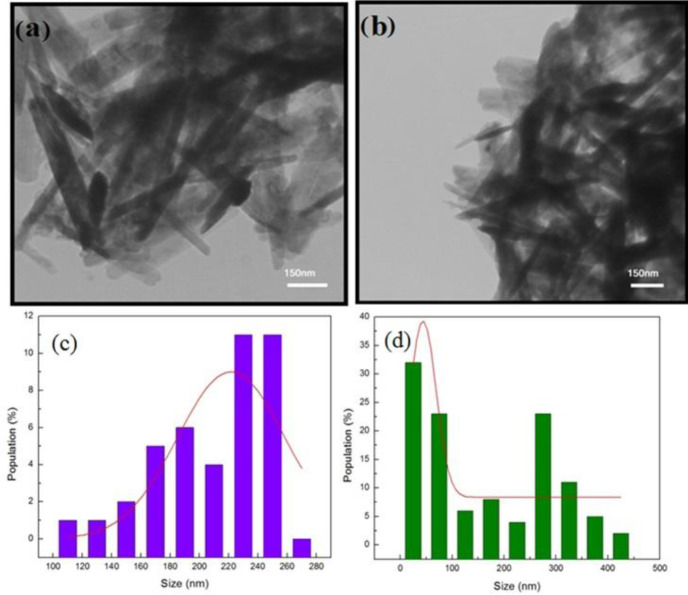
TEM images from aggregation mode of HNRs prepared with (a) 3.2 mg/ml and (b) 6.4 mg/ml of albumin on a scale of 150 nm. The size distribution of HNRs prepared in (c) 3.2 mg/ml and (d) 6.4 mg/ml concentration of albumin

TEM: Transmission electron microscopy; HNRs: Hematite nano rods

TEM interaction images of HNRs with higher concentrations of albumin showed changes in the morphology (Figure 3) and aggregation mode of samples (Figure 4, a-b). This process was done due to increasing protein concentration relative to CTAB. Consequently, the magnetic nanorods contain CTAB and lose their rod structure (37-40). Figure 4 (c, d) shows that the average length of magnetic nanorods was reduced by increasing the concentration of albumin. 

*VSM of HNR*

**Figure 5:**
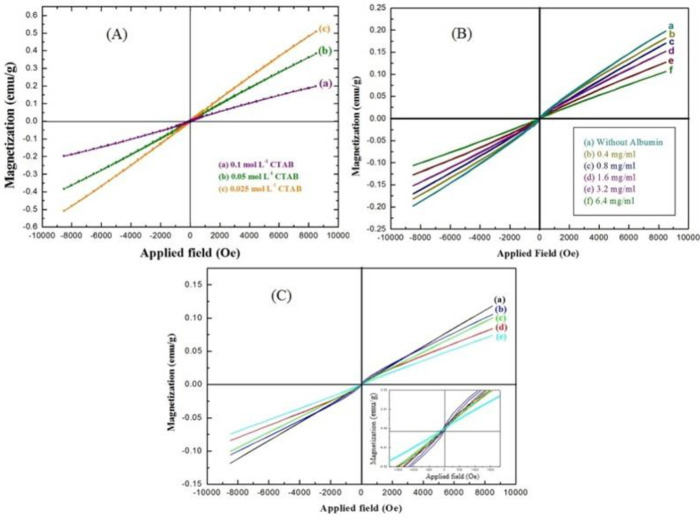
(A) Magnetic hysteresis curves of HNRs prepared with (a) 0.1 mol L^-1^, (b) 0.05 mol L^-1^, and (c) 0.025 mol L^-1 ^of CTAB. (B) Magnetic hysteresis curves of HNRs prepared in interaction with albumin with concentration of (a) without albumin, (b) 0.4 mg/ml, (c) 0.8 mg/ml, (d) 1.6 mg/ml, (e) 3.2 mg/ml, and (f) 6.4 mg/ml. (C) Magnetic hysteresis curves of HNRs with 0.1 mol L-1 of CTAB in (a) 0.4 mg/ml, (b) 0.8 mg/ml, (c) 1.6 mg/ml, (d) 3.2 mg/ml, and (e) 6.4 mg/ml of albumin

HNRs: Hematite nano rods; CTAB: Cetyl trimethylammonium bromide

VSM analysis is generally used to introduce a specific magnetization and a certain range of concentrations of albumin in quantitative assays to identify the amount of protein in the urine. Figure 5A shows the hysteresis curve of HNR prepared in different molarities of CTAB between -10000 and 10000 Oersted (Oe). The VSM spectrum of HNRs showed that magnetization of HNRs reduced by increasing the concentration of CTAB due to formation of more layers of surfactant agent on the surface of HNRs (41). Figure 5B shows magnetic hysteresis curves of HNRs prepared through interacting with albumin at different concentrations. There were no considerable variations in the numerical value of the magnetization between samples without albumin and with 0.4 and 0.8 mg/ml of albumin, but at other concentrations of albumin, there was a difference in magnetization between them. The magnetic characteristics of the samples showed that higher magnetization of HNRs can be observed for samples with less concentration of CTAB and albumin. This spectrum determines the sensitivity of the above method to differentiate these values from each other. Magnetic characteristics of HNRs-albumin were measured at room temperature (RT). Figure 5C shows the hysteresis curves of the HNRs-albumin-CTAB samples at constant concentrations of CTAB and variable concentrations of albumin. The inset of Figure 5C demonstrates the VSM graph of samples with anti-ferromagnetic behavior at a low applied field of -1800 Oe. The maximum and minimum numerical values of hydrothermally synthesized nanostructures were equal to 0.11 emu/g and 0.07 emu/g. \

(B): UV–vis spectra of HNR prepared with: (a) without CTAB, (b) 0.025 mol L^-1^, (c) 0.05 mol L^-1^, and (d) 0.1 mol L^ -1^ CTAB at RT. (C): UV–vis spectra of HNR prepared with 0.025 mol L^-1^ of CTAB in (a) 15 ml DIW, (b) 10 mL DIW, and (c) 5 ml DIW at RT. (D): UV–vis spectra of HNR prepared with (a) 0.4 mg/ml, (b) 0.8 mg/ml, (c) 1.6 mg/ml, (d) 3.2 mg/ml, and (e) 6.4 mg/ml albumin solution at RT

CTAB: Cetyl trimethylammonium bromide; HNR: Hematite nano rod

TEM: Transmission electron microscopy

**Figure 6:**
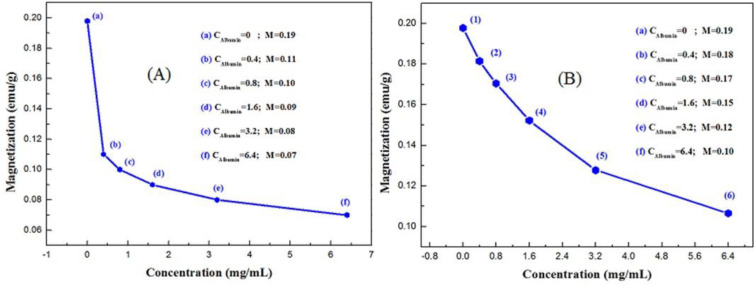
(A) Magnetization curve in terms of concentration for HNRs in interaction with albumin prepared with (a) A:C _0.1_, (b) A:B_0.4_:C_ 0.1_, (c) A:B_0.8_:C _0.1_, (d) A:B_1.6_:C_0.1_, (e) A:B_3.2_:C_ 0.1_, and (f) A:B_6.4_:C _0.1_. (B) Magnetization curve in terms of concentration for HNRs in interaction with albumin prepared with (a) A:C _0.0625_, (b) A:B_0.4_:C _0.0125_, (c) A:B_0.8_:C _0.025_, (d) A:B_1.6_:C_0.05_, (e) A:B_3.2_:C _0. 1_ and (f) A:B_6.4_:C _0. 2_

A=Fe_3_O_4_, B= albumin (C_123_H_193_N_35_O_37_) and C=CTAB (C_19_H_42_BrN)

HNRs: Hematite nano rods; CTAB: Cetyl trimethylammonium bromide

**Figure 7:**
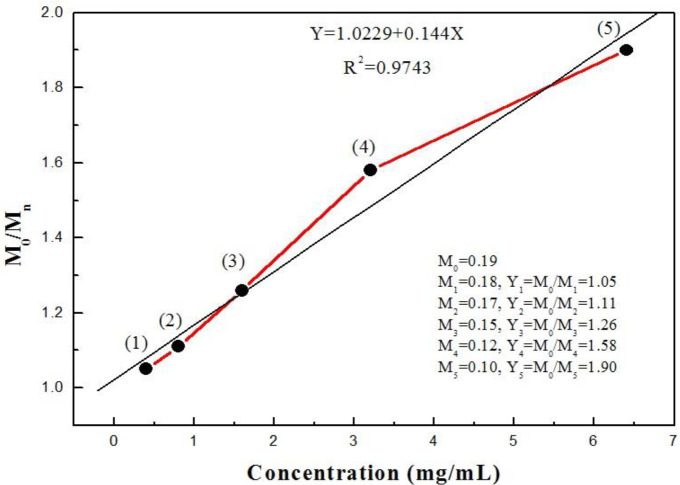
Standard curve of HNRs and line coefficient repeatability (R2=0.9743)

HNRs: Hematite nano rods

**Figure 8:**
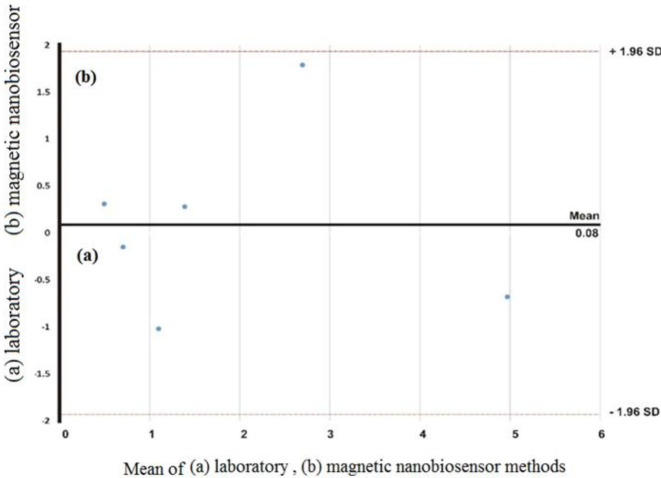
Bland-Altman plot for measuring albuminuria (mg/ml) with (a) laboratory and (b) magnetic nanobiosensor methods

## Discussion

*Determining the amount of albuminuria with HNR nanobiosensor*

When kidneys get stuck or blocked, urine volume reduces below that of healthy people. The amount of protein in the patient’s urine volume is condensed which is a dangerous biomarker and indicates the destruction of kidney cells (42-43). In laboratory methods, urine protein will be measured in the 24-hr or 12-hr mode (44). If the concentration of protein in this volume of urine is greater than a certain amount, it shows dangerous levels of albumin in the urine (45-46). The goal is to design a nanobiosensor that can detect in a lower normal range of urine protein (47). Therefore, if the minimum amount of protein is detected in the urine, it has a higher sensitivity and shows kidney damage sooner. In designing a magnetic nanobiosensor, sensitivity and repeatability are two important parameters that are important to us in rapidly diagnosing a disease. In this regard, we need to point out that distinction between the current work and previous research is the use of magnetic nanorod for higher magnetization and increased contact with biomolecules (48-49). The results of the clinical performance are shown in the standard curve and Bland-Altman plot. By studying relevant research, we find that most biological interactions are between the albumin protein and the magnetic nanoparticles that have been introduced in therapeutic areas such as hyperthermia (50). So, considering that the diagnosis is prior to treatment and the importance of the role of magnetic nanorods in medicine (51), it is possible to find out the effectiveness of kits made of magnetic nanorods for the diagnosis of kidney disease. In parallel with the above research, Megan Casco and *et al*. used standard curves of iron nanoparticles in interacting with cells to stimulate extra cells and produce matrix in spleen cells and deformation of nanoparticles using magnetic nanorod to produce an extracellular matrix with effect on the shape of nanoparticles (52).

Figures 6A and 6B show the magnetization curve for Fe_3_O_4_ nanorods at 0, 0.4, 0.8, 1.6, 3.2, and 6.4 mg/ml concentration of albumin (C_123_H_193_N_35_O_37_) in constant and variable concentrations of CTAB (C_19_H_42_BrN), respectively. As we expect, in both graphs we see that the interaction of albumin protein with CTAB reduced magnetization, proving the presence of albumin in the sample. Amounts of albumin in biological samples were obtained with quantitative data from the magnetization curve in terms of concentration. So, we succeeded in designing this as a qualitative assay and then we can quantitative using the standard curve. We need to make a linear regression from the points obtained in the magnetized curve in terms of concentration. The magnetization of each of the points is related to the concentration. Figure 7 shows the standard curve of HNRs interacting with albumin. With increasing concentrations of albumin in presence of CTAB, magnetization shows rather little variation of the magnetism with large variations of the albumin concentration. The quantitative measurement of protein was obtained through the association between magnetization and the standard range of albumin concentration. As a result, it could be claimed that the method was sensitive enough to detect urinary albumin. It would be sufficient just to calculate the ratio of magnetization in non-interaction samples (M_0_) to interaction samples (M_n_) to test clinical samples with unknown concentrations and certain magnetization. The numerical value of concentration was obtained by putting this ratio in the standard curve in which R^2 ^was considered the repeatability coefficient line. Based on the relationship between magnetization (Y= M_0_/M_n_) and concentration (X) of albumin, the linear regression equation is obtained in the form of Y=1.0229+0.144X. M_0_/M_n_ ratio was measurable according to this formula. The numerical value of the repeatability coefficient (R^2^) is 0.9743. Therefore, the result obtained from the standard equation indicates the high reproducibility of the HNR nanobiosensor in accurate identification of albuminuria. We observed a linear relationship between magnetization and albumin concentration in the standard range. 

In fact, accuracy and precision are very important to represent the correct detection rate of the test. Accuracy does not always mean precision and vice versa. The ideal mode is to have accuracy and precision together. **Table 1** is a comparison between the amount of albuminuria obtained in the laboratory and by the HNR biosensor method. Figure 8 shows the Bland-Altman statistical diagram (53) for comparing the two *in vitro* and HNR biosensor methods. In the Bland-Altman plot, the middle line was the mean value, and the two lines around the mean value represent the **±**1.96 SD values. The average in this chart was at the zero point. The set of points that were in the positive and negative range of deviation from the standard were a set of acceptable answers. The closer the points were to the mean line, the higher the accuracy of the method. As a result, the standard curve of magnetic nanosensors with 97% accuracy and repeatability was able to detect the lowest level of microalbuminuria (MAU) in clinical samples. In addition, the results of the Bland-Altman statistical data showed that magnetic biosensors, like the experimental methods for albumin detection, have repeatability and reliability.

## Conclusion

HNRs were synthesized using the hydrothermal method with CTAB as a surfactant agent at different molarities. We obtained a standard equation through interaction between HNRs and albumin for early detection of albuminuria. This magnetic nanobiosensor could detect even the least amount of microalbumin in the urine. The results obtained from clinical samples showed that the HNR biosensor with 97% accuracy was capable of detecting albuminuria within the 0.4 mg/ml to 6.4 mg/ml standard range.
